# Expression of Activated *PIK3CA* in Ovarian Surface Epithelium Results in Hyperplasia but Not Tumor Formation

**DOI:** 10.1371/journal.pone.0004295

**Published:** 2009-01-27

**Authors:** Shun Liang, Nuo Yang, Yue Pan, Shan Deng, Xiaojuan Lin, Xiaojun Yang, Dionyssios Katsaros, Katherine F. Roby, Thomas C. Hamilton, Denise C. Connolly, George Coukos, Lin Zhang

**Affiliations:** 1 Center for Research on the Early Detection and Cure of Ovarian Cancer, University of Pennsylvania School of Medicine, Philadelphia, Pennsylvania, United States of America; 2 Department of Obstetrics and Gynecology, University of Pennsylvania School of Medicine, Philadelphia, Pennsylvania, United States of America; 3 Abramson Family Cancer Research Institute, University of Pennsylvania School of Medicine, Philadelphia, Pennsylvania, United States of America; 4 Department of Obstetrics and Gynecology, University of Turin, Turin, Italy; 5 Center for Reproductive Sciences, University of Kansas Medical Center, Kansas City, Kansas, United States of America; 6 Ovarian Cancer Program, Fox Chase Cancer Center, Philadelphia, Pennsylvania, United States of America; University of Hong Kong, Hong Kong

## Abstract

**Background:**

The Phosphatidylinositol 3′-kinase is a key regulator in various cancer-associated signal transduction pathways. Genetic alterations of its catalytic subunit alpha, *PIK3CA*, have been identified in ovarian cancer. Our *in vivo* data suggests that *PIK3CA* activation is one of the early genetic events in ovarian cancer. However, its role in malignant transformation of ovarian surface epithelium (OSE) is largely unclear.

**Methodology/Principal Findings:**

Using the Müllerian inhibiting substance type II receptor (MISIIR) promoter, we generated transgenic mice that expressed activated *PIK3CA* in the Müllerian epithelium. Overexpression of *PIK3CA* in OSE induced remarkable hyperplasia, but was not able to malignantly transform OSE *in vivo*. The consistent result was also observed in primary cultured OSEs. Although enforced expression of *PIK3CA* could not induce OSE anchorage-independent growth, it significantly increased anchorage-independent growth of OSE transformed by mutant *K-ras*.

**Conclusions/Significance:**

While *PIK3CA* activation may not be able to initiate OSE transformation, we conclude that activation of *PIK3CA* may be an important molecular event contributing to the maintenance of OSE transformation initiated by oncogenes such as *K-ras*.

## Introduction

Epithelial ovarian cancer continues to be the leading cause of death among gynecological malignancies [1], [2], [3]. The lack of effective methods for prevention, early detection and treatment recurrent ovarian tumors creates a pressing need to understand its pathogenesis and identify molecular targets for therapy. Cancer is a disease involving multistep dynamic changes in the genome [4]. However, the oncogenic events and their cooperation that promote malignant transformation in ovarian carcinoma remain largely unknown [2], [3], [5], [6], [7], [8], [9], [10].

Phosphatidylinositol-3′ kinase (PI-3 kinase) is an intracellular signal transducer with lipid substrate specificity implicated in a wide range of cancer-associated signaling pathways including tumor cell metabolism, survival and proliferation [11], [12], [13], [14], [15], [16], [17], [18], [19]. It is recruited and activated by multiple receptor tyrosine kinases and generates second messengers via phosphorylation of membrane inositol lipids at the D3 position [11], [12], [13], [14]. PI-3 kinase was first recognized as a putative oncogene because of its ability to bind polyoma middle T antigen [20], [21]. Molecular cloning of PI-3 kinases revealed a large and complex family that contains three classes with multiple subunits and isoforms [11], [12], [13], [14]. The *PIK3CA* gene encodes the catalytic subunit p110-alpha, one of the three catalytic subunit proteins of the class IA PI-3 kinases [11], [12], [13], [14]. *PIK3CA* was identified as an avian retrovirus-encoded oncogene that can transform chicken embryo fibroblasts [22]. Numerous recent studies indicate that *PIK3CA* and downstream pathways are frequently targeted by genomic amplification [23], mutation [24] or overexpression [25] in solid tumors including ovarian cancer [23], [25], [26], [27], [28], [29], [30], [31], [32]. Previous studies on the function of *PIK3CA* in ovarian cancers have been predominantly focused on the maintenance and survival of late-stage of ovarian carcinoma. The function of *PIK3CA* in malignant transformation of the ovarian surface epithelium (OSE) remains unexplored. Here we generated transgenic mice expressing constitutively activated (myristoilated) *PIK3CA* in the Müllerian epithelium of the female genital tract to investigate the effect of *PIK3CA* overexpression in the OSE.

## Results

### *PIK3CA* overexpression was an early genetic event during ovarian tumorigenesis

**Figure 1:**
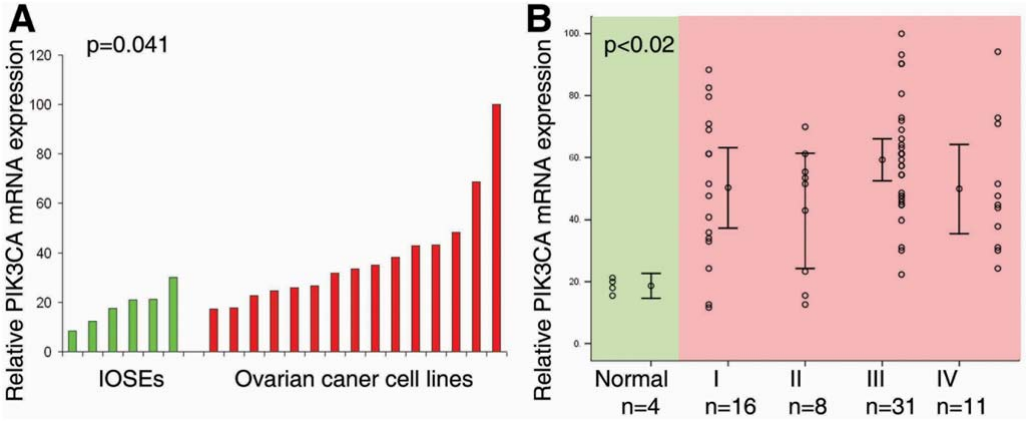
*PIK3CA* overexpression was an early genetic event during ovarian tumorigenesis. A. *PIK3CA* mRNA expression was significantly up-regulated in the established epithelial ovarian cancer cell lines (n = 15) compared with primary cultured ovarian surface epithelial cells (n = 6, p = 0.041). mRNA expression was measured by real-time RT-PCR. B. mRNA expression level of *PIK3CA* was significantly upregulated in ovarian cancer specimens compared with normal control ovarian epithelium. Normal ovarian epithelium was isolated by laser-capture microdissection. There was no further significant increase of *PIK3CA* mRNA expression after malignant transformation among different stages of ovarian cancer.

**Figure 2:**
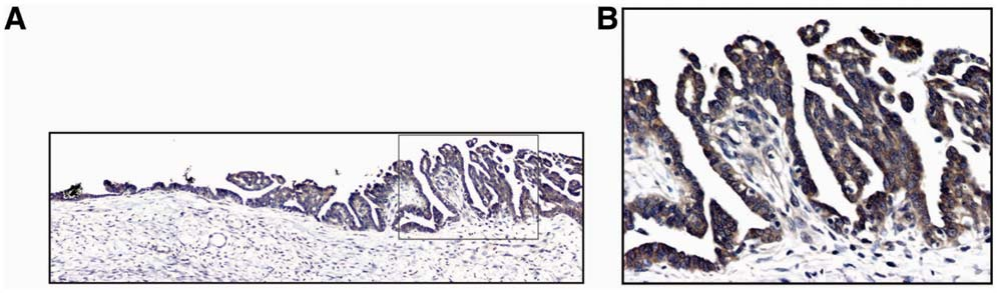
High p110α expression was detected in early malignant transformed human ovarian surface epithelium. p110α expression was detected by immunohistochemical staining. A. High p110α expression was detected in early malignant transformed human ovarian surface epithelium (100X). B. High magnification of A (400X).

In many human tumors, including epithelial ovarian cancer [23], [25], [26], [27], [28], [29], [30], *PIK3CA* activation is a critical oncogenic event and can be mediated by multiple genetic/genomic alterations such as gene copy number amplification [23], gain of functional mutations [24], and transcriptional up-regulation [33], [34], [35]. However, the function of *PIK3CA* activation during the process of malignant transformation of OSE is still not well understood. Our previous studies indicate that mRNA expression of *PIK3CA* is significantly up-regulated in the early-stage of ovarian cancer development, strongly suggesting that *PIK3CA* might be involved in OSE transformation [25]. To confirm our previous finding, we first compared expression levels of *PIK3CA* mRNA in established epithelial ovarian cancer cell lines (n = 15) with primary cultures of immortalized human ovarian surface epitheliums (IOSEs, n = 6). Consistently, we found that mRNA levels in epithelial tumor cell lines were significantly higher than in IOSEs (p = 0.041, Figure 1A). Next, we examined mRNA expression of *PIK3CA* in microdissected normal human ovarian epithelium (n = 4) as well as epithelial ovarian cancer specimens including FIGO stages I (n = 16), II (n = 8), III (n = 31) and IV (n = 11). We found that mRNA expression level of *PIK3CA* was significantly upregulated in ovarian cancer specimens compared to normal control ovarian epithelium (p<0.02), and there was no further significant increase after malignant transformation among different stages of ovarian cancer (p>0.05, Figure 1B), which is consistent with our previous observation [25]. To further confirm *PIK3CA* is indeed expressed in early-stage ovarian cancer, we also examined the protein product of *PIK3CA* gene, p110α, by immunohistochemical staining in early malignant transformed human ovarian surface epithelium. We found that p110α was highly detectable in the early malignant transformed human ovarian surface epithelium (Figure 2A and B). Taken together, these results indicate that *PIK3CA* overexpression is in fact an early genetic event during ovarian oncogenesis, thus suggesting that *PIK3CA* activation might be causally involved in this process.

### Generation of transgenic mice expressing activated *PIK3CA* in the Müllerian epithelium

**Figure 3:**
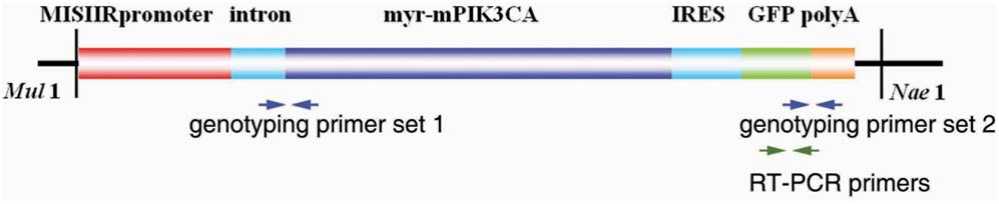
The illustration of the construct that was used to generate transgenic mouse. The arrows show two sets of the genotyping primers and one set of the RT-PCR primers.

**Figure 4:**
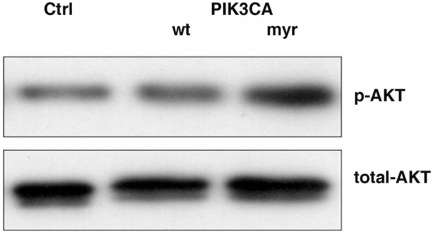
*myr-PKI3CA* was able to active PI3-kinase pathway. myr-*PIK3CA*, wt-*PIK3CA* and control vectors were transient transfected to ovarian cancer cell line 2008. 48 hrs of post-transfection, transfected cells were cultured in 1% serum over night. Total and phosphate AKT were detected by western blot.

To investigate the role of *PIK3CA* in malignant transformation of the OSE *in vivo*, we generated transgenic mice in which activated *PIK3CA* was specifically overexpressed in the Müllerian epithelium of the female reproductive tract including OSE. In this model, we used the Müllerian epithelium specific promoter, Müllerian inhibiting substance type II receptor promoter (*MISIIR*) [36], [37], [38], [39], to drive expression of the murine *PIK3CA* (Figure 3). In the male animal, Müllerian inhibiting substance (*MIS*) is secreted from Sertoli cells of the developing testes and stimulates the regression of the Mullerian duct. Testosterone is also secreted from the developing testes and induces the differentiation of the Wolffian duct into the secondary structures of the male reproductive tract. In the absence of MIS in the developing female embryo, the Mullerian duct differentiates into the secondary structures of the female reproductive tract [36], [37], [38], [39]. Expression of the *MISIIR* has been reported to be restricted to mesenchymal cells surrounding the Mullerian duct during embryogenesis, tubular and follicular structures of fetal gonads, Sertoli and Leydig cells of adult testis, and granulosa cells of adult ovary [36], [37], [38], [39]. Above information provides a possible strategy to develop a transgenic model of ovarian carcinoma [39]. Using this promoter, Connolly *et al*. have successfully developed the very first ovarian cancer transgenic models that develop ovarian carcinomas with metastatic spread to peritoneal organs [39]. In addition, increasing evidence indicates that *PIK3CA* is activated in a large percentage of human ovarian cancer patients [25], [27], [35]. Therefore, we generated the activating mutation by addition of the avian src myristoylation sequence (MGSSKSKPK) at the N-terminus of the wild type of murine *PIK3CA* to constitutively activate PI3-kinase pathway *in vivo*. To demonstrate that our transgenic construct was able to constitutively activate PI3-kinase pathway, we transient transfected myr-*PIK3CA*, wt-*PIK3CA* and control vectors to ovarian cancer cell line 2008. 48 hrs of post-transfection, the transfected cells were cultured in low serum overnight. Protein and total RNA were isolated from cells. Real-time RT-PCR demonstrated that cells from wt-*PIK3CA* and myr-*PIK3CA* transfections were expressed similar levels of *PIK3CA* mRNA, which was about 11.5-fold higher comparing to cells from control vector transfection. Total and phosphate AKT, the downstream molecule of PI3-kinase pathway, were examined by western blot. Figure 4 showed that myr-*PIK3CA* was able to constitutively activate AKT in low serum condition (1%) compared with wt-*PIK3CA* and control transfection.

**Figure 5:**
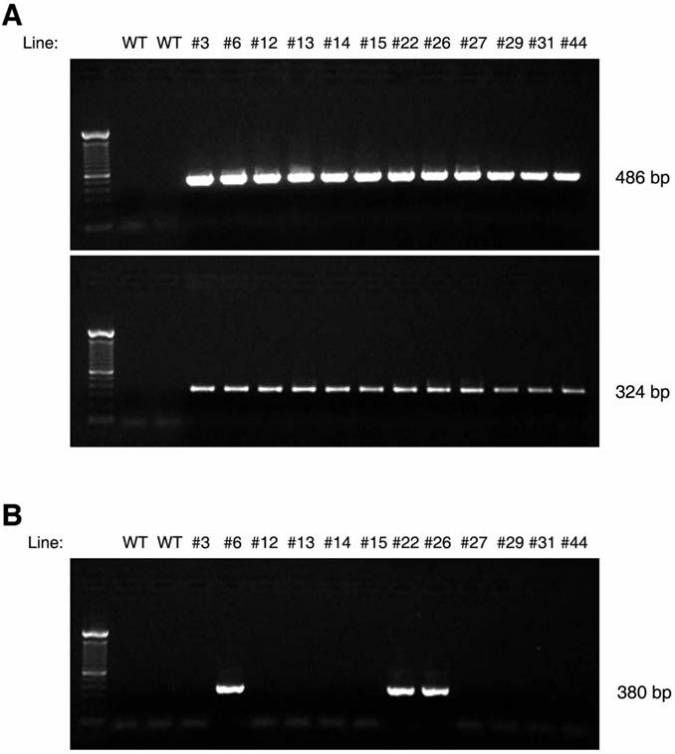
Generation of transgenic mice expressing activated *PIK3CA* in the Müllerian epithelium. A. A total of 12 founder lines were generated. Upper panel shows the genotyping by PCR using the first set of the genotyping primers. Lower panel shows the genotyping by PCR using the second set of the genotyping primers. B. The transgene (eGFP) was able to be detected in 3 founder lines by RT-PCR.

Twelve founder lines of MISIIR-*PIK3CA* transgenic (*PIK3CA*-Tg) mice were generated, in which the genomic integration of whole MISIIRpr-myr*PIK3CA*-IRES-GFP sequence was confirmed by two sets of genotyping primers (Figure 5A). Expression of the transgene mRNA (eGFP) was examined by RT-PCR in the samples from the female whole ovary of the genotype-positive mice. In three of the transgenic lines, the eGFP mRNA transcript was able to be detected *in vivo* (Figure 5B). Three founder *PIK3CA*-Tg lines (#6, #22 and #26), three control lines (genotype positive, eGFP mRNA negative #3, #12 and #44, Figure 5B) and wild type mice (genotype negative mice) were used for further studies.

### Overexpression of activated *PIK3CA* in OSE resulted in hyperplasia but not tumor formation

**Figure 6:**
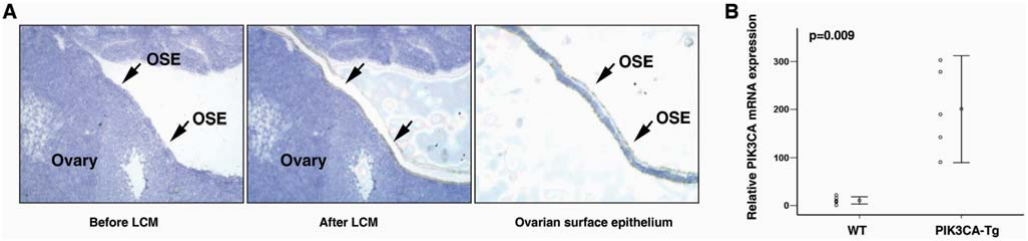
*PIK3CA* was overexpressed in OSE of *PIK3CA-Tg* mouse. A. Ovarian surface epithelial cells were microdissected by laser capture microdissection technology. B. *PIK3CA* mRNA expression in OSEs from WT or *PIK3CA-Tg* OSEs was analyzed by real-time RT-PCR. The primers were able to detected both wt *PIK3CA* cDNA and cDNA from transgene expression (myr-*PIK3CA*).

**Figure 7:**
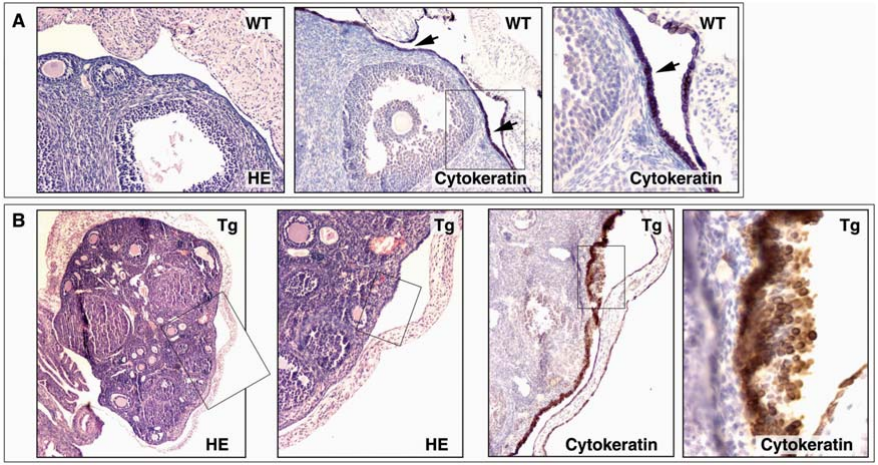
Overexpression of activated *PIK3CA* in OSE resulted in hyperplasia but not tumor formation. A. The ovarian surface epithelium in wild type mouse. B. The ovarian surface epithelium in *PIK3CA*-Tg mouse. The epithelium origin was confirmed by cytokeratin staining.

To confirm overexpression of *PIK3CA* in the OSEs of transgenic mouse, we microdissected the OSE cells from *PIK3CA*-Tg and WT mice using laser capture microdissetion (LCM, Figure 6A). Total RNA was isolated, and *PIK3CA* mRNA expression was measured by real-time RT-PCR. We found that *PIK3CA* mRNA expression level was 18.5-fold higher in OSEs from *PIK3CA*-Tg mice (200.93 ±89.47 relative expression unit) than from WT mice (10.87 ± 7.28 relative expression unit, p = 0.009, Figure 6B). In addition, by immunohistochemistry (using two different antibodies), we confirmed that p110α was highly expressed in the OSE of the *PIK3CA*-Tg mice (data not shown). Then, we followed up the ovarian tumor development in *PIK3CA*-Tg mice. We found that expression of the activated *PIK3CA* resulted in hyperplasia in mouse OSE and a paucity of follicles in four month-old female mice. Female transgenic *PIK3CA*-Tg mice exhibited subfertility. Figure 7A shows the typical OSE in 5-month control female mice. The Cytokeratin (an epithelial marker) positive OSE was observed on the surface of ovary as a monolayer. In the *PIK3CA*-Tg mice, hyperplasia was found in the OSE, and the epithelial origin of the lesions was confirmed by Cytokeratin staining (Figure 7B). Hyperplasia was found in more than 50% of the *PIK3CA*-Tg mice after four months post-birth, and in 100% of the *PIK3CA*-Tg mice after ten months post-birth. There were no invaginations or papillary structures observed in the OSE of *PIK3CA*-Tg mice. In the control mice, no significant hyperplasia in OSE was observed even after 12 months of post-birth. These results suggest that expression of activated *PIK3CA* in OSE induced hyperplasia of the OSE. We monitored for ovarian tumor development in both the transgenic and control mouse lines. A total of 218 female *PIK3CA*-Tg mice (#6: n = 94; #22: n = 44 and #26: n = 80) were evaluated (at least 30 mice of each line were followed for more than 18 months) and no epithelial ovarian tumors were observed. There was no difference in life span between the transgenic mice and control lines. This finding indicates that expression of activated *PIK3CA* in OSE causes hyperplasia *in vivo* but not tumor formation.

### *PIK3CA* contributed to *K-ras* initiated transformation *in vitro*

**Figure 8:**
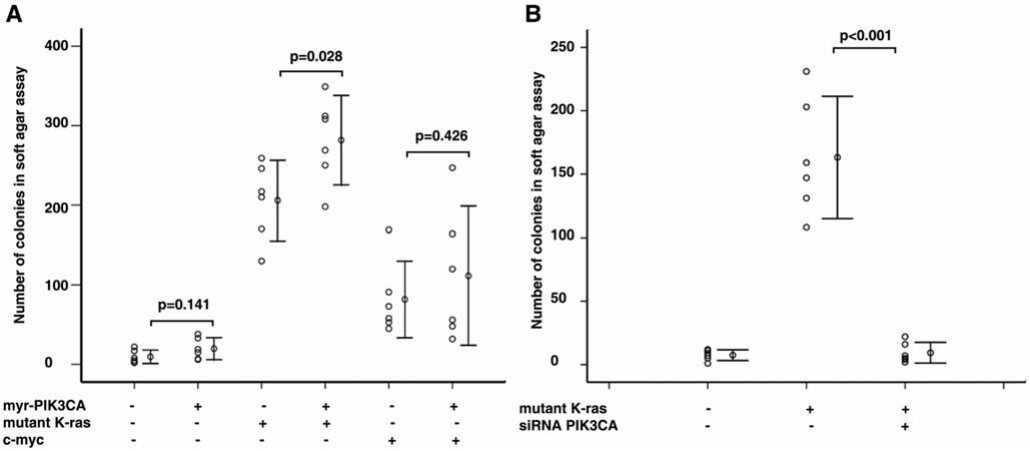
*PIK3CA* contributed to *K-ras* initiated transformation *in vitro.* A and B. Summary of colony numbers of the different combination of oncogenes in soft agar assay.

To further confirm the above *in vivo* observation, we next tested the role of *PIK3CA* in OSE transformation *in vitro*. Primary murine OSE cells (MOSEs) were isolated from ovaries of female mice and cultured short-term (only early passage, <10 passages, cells were used in this study) [40]. Soft agar assay was used to evaluate the potential contribution of *PIK3CA* in transformation of OSE [41]. Because PI3-kinase/PTEN-ras [8] and PI3-kinase/AKT-myc [5] pathways have been reported involving in OSE transformation, we chose ras and c-myc in our following studies. Myr-*PIK3CA* (pUSEamp-myr-m*PIK3CA*, under CMV promoter, G418), K-ras^v12^ and c-myc (both in pBabe-puro vector, puromycin) expression vectors were transfected to MOSE cells. The pooled stable expression cells were generated by short-term antibiotic selection (10 days for G418 or 3 days for puromycin). The transgene expression was further confirmed by RT-PCR. We found that overexpression of *PIK3CA* alone did not significantly increase anchorage-independent growth of OSEs (Figure 8A). In contrast, introduce of either mutant *K-ras* of *c-myc* resulted in increased colony formation of OSEs (Figure 8A). This result indicates that unlike mutant *K-ras* or *c-myc*, *PIK3CA* is not able to cause complete transformation of OSE *in vitro*. Next, we tested the combination of *PIK3CA* with either of the two other oncogenes by co-transfection. The myr-*PIK3CA* was transfected first. After 10 days G418 selection, *K-ras* or *c-myc* was transfected subsequently and selected again by puromycin for 3 days. Interestingly, we found that combining *PIK3CA* and mutant *K-ras* significantly increased anchorage-independent growth of cultured OSE cells compared with mutant *K-ras* alone (Figure 8A). However, *PIK3CA* did not significantly increase colony numbers of OSE cells transformed by *c-myc* (Figure 8A). This finding suggests that early activation of *PIK3CA* might promote transformation of OSE cells in certain cellular and molecular contexts, e.g. in the presence of *K-ras* mutation. To further confirm that *PIK3CA* can play a role in transformation induced by mutant *K-ras*, we blocked endogenous *PIK3CA* expression in cultured OSE cells transfected with mutant *K-ras* by RNA interference using small interfering RNAs (siRNA). The efficiency of the siRNA targeting *mPIK3CA* was confirmed by real-time RT-PCR (mRNA expression of *mPIK3CA* was knocked down to ∼30% in the siRNA treated cells compared with control cells, data not shown). The specificity of the siRNA was also examined by measuring endogenous *mPIK3CB* expression (there was no significantly difference of *PIK3CB* mRNA expression between siRNA treated and control cells, data not shown). We found that in the absence of *PIK3CA*, the transformed OSEs could not grow in soft agar (Figure 8B), which suggests that the growth of *K-ras* transfected OSE cells require *PIK3CA* expression. However, it is still unclear whether *PIK3CA* contributes to *K-ras* initiated transformation *in vivo.* Generation of “bigenic” mouse expressing both activated *PIK3CA* and mutant *Ras* specifically in murine OSE using *MISIIR* promoter is still a technical challenge. Because mutant *Ras* is able to fully transform epithelium *in vivo* [42], *MISIIR-*driven mutant *Ras* will induce very rapidly reproductive system carcinoma in both female and male animals, and these transgenic mice will lose reproductive ability. Therefore, it will be difficult to generate “bigenic” animals by crossing *MISIIR-*driven mutant *Ras* and *MISIIR-*driven myr-*PIK3CA* mice. We believe that a novel transgenic strategy based on the Cre-loxp conditional expression and intrabursal administration of adenovirus [6], [8], [9] may allow us to further test *PIK3CA* and *Ras* cooperation *in vivo* in the future.

## Discussion

In agreement with our findings, several groups have demonstrated that activation of PI3-kinase plays a crucial role in epithelial cell transformation. For example, in human mammary epithelial cells (HMECs), activation of the PI3-kinase pathway in the presence of increased expression of *c-myc*, can functional replace small T antigen and result in anchorage-independent growth [43]. In p53 null murine OSE cells, enforced expression of the PI3-kinase downstream mediator, *Akt*, in cooperation with mutant *K-ras,* induces the OSE cell transformation [5]. Moreover, two independent laboratories have recently reported that conditional *Pten* deletion combined with mutant *K-ras* [8] or deregulated *Wnt/Catenin* pathway [9] is able to induce endometrioid ovarian tumors in mice *in vivo*. In all of these studies, only activation of wild type *PIK3CA* alone seems insufficient to initiate epithelial transformation, consistent with the results of our study. Although mutant *PIK3CA* has been reported to be sufficient to transform normal cells both *in vitro* [44], [45], [46] and *in vivo* [46], [47], *PIK3CA* mutation exhibits a relatively low frequency in ovarian cancer (4.8–12%) [28], [29], [30], [32] as compared to other cancer types. In addition, *PIK3CA* mutation is rare in borderline [30] or early-stage ovarian tumors (our unpublished observation). Therefore, *PIK3CA* mutation might not be commonly involved in the transformation of the OSE. However, *PIK3CA* amplification is one of most common genetic alterations in ovarian cancer (23.6–40%) [23], [26], [27], [31] and more importantly, an increase in copy number at chromosomal locus 3q26-qter, which harbors the *PIK3CA* gene, has been observed in 40% of early-stage ovarian cancers [26], suggesting that *PIK3CA* amplification might be one of the critical events in OSE transformation. In this study, we tested overexpression of activated wild type *PIK3CA* in OSE *in vivo* and *in vitro*, since it might more closely resemble natural OSE transformation during human ovarian cancer development. Taken together, we conclude that *PIK3CA* activation is one of the early molecular events during OSE transformation, and activation of *PIK3CA* contributes to tumorigenesis in certain cellular and molecular contexts. However, *PIK3CA* activation might not be the initial event in OSE transformation, and may require cooperation with other oncogenic events such as *K-ras* mutation to maintain transformed OSE growth.

## Materials and Methods

### Patients and Specimens

The specimens used in this study were collected at the University of Pennsylvania and the University of Turin, Italy. All tumors were from primary sites, and were immediately snap-frozen and stored at −80°C. Ethical approval for this work was granted by institution's Institutional Review Boards (IRBs) of the University of Pennsylvania and the University of Turin. Tissues were obtained after informed written consent from patients involved under a general tissue collection protocol approved by the IRBs.

### Cell lines and Cell Culture

A total of 15 ovarian cell lines were used in this study. All cancer cell lines were cultured in DMEM medium (Invitrogen, Carlsbad, CA) supplemented with 10% fetal bovine serum (FBS, Invitrogen). Six independent immortalized human ovarian surface epithelial cells (IOSEs, generously provided by Drs. Auersperg and Birrer) were cultured in 1∶1 media 199: MCDB 105 (Sigma, St. Louis, MO) supplemented with 15% FBS. Murine ovarian surface epithelial cells (MOSEs) were isolated and cultured as previously reported [40].

### Plasmid Construction

pMISIIRprom-TOPO, which contains the murine Müllerian inhibitory substance type II receptor (MISIIR) gene 5′ regulatory region, was constructed by PCR amplification from mouse genomic DNA [39]. pUSEamp-myr-m*PIK3CA*, which contains murine *PIK3CA* under the control of CMV promoter, was ordered from UPSTATE (UPSTATE, Lake Placid, NY). The activating mutation was generated by addition of the avian src myristoylation sequence (MGSSKSKPK) at the N-terminus. pCI-neo, which contains a chimeric intron, was ordered from Promega (Promega, Madison, WI). pMigR, which contains an internal ribosome entry site (IRES) and downstream enhanced green fluorescent protein (eGFP), was generously provided by Dr. Pear. We tagged myr-*PIK3CA* in pUSEamp with eGFP, preceded by an IRES. The IRES and eGFP were derived from pMigR. The *XhoI-SalI*-blunted fragment containing the IRES and eGFP from plasmid pMigR was inserted into the *XhoI-ApaI*-blunted plasmid pUSEamp-myr-*PIK3CA* to construct plasmid pUSEamp-myr-*PIK3CA*-IRES-GFP. The *SacI-XhoI* linearized fragment containing the chimeric intron from plasmid PCI-neo was inserted into the *EcoRV-XhoI* linearized plasmid pMISIIRprom-TOPO to construct plasmid pMISIIRprom-Intron. The MluI-*ScaI* linearized fragment containing the *MISIIR* promoter and the intron from plasmid pMISIIRprom-Intron was swapped into plasmid pUSEamp-myr-*PIK3CA*-IRES-GFP to make plasmid pMISIIRprom-Intron-myr-*PIK3CA*-IRES-GFP, in which the CMV promoter has been replaced by the *MISIIR* promoter and the intron upstream of the *PIK3CA* ORF, as well as the IRES, eGFP reporter and hGH polyA downstream (for the detailed information for construction also see Figure S1). This construct was used to generate a transgenic mouse expressing myr-*PIK3CA* in the OSE.

### Protein Isolation and Western blot

Cultured cells were lysed in 200 μl of lysis buffer containing 50 mM Tris-HCl (pH 7.4), 150 mM NaCl, and 1% Triton X-100. Protein was separated by 10% SDS-PAGE under denaturing conditions and transferred to nitrocellulose membrane. Membranes were incubated with an anti-total AKT or anti-pAKT antibodies (1∶1,000, Cell Signaling Technology, Danvers, MA), followed by incubation in anti-rabbit secondary antibody conjugated with horseradish peroxidase (1∶10,000; Cell Signaling Technology). Immunoreactive proteins were visualized using enhanced chemiluminescence detection system (Amersham Biosciences, Piscataway, NJ).

### Transgenic Mice

The animal study protocol was reviewed and approved by the institutional animal care and use committee (IACUC) of the University of Pennsylvania. Transgenic mice were generated by the University of Pennsylvania's Transgenic & Chimeric Mouse Facility. The linearized transgene DNA fragment (*MISIIR-myr-PIK3CA-IRES-GFP*) was injected by microinjection into pronuclei of day-0.5 embryos of the first generation of a hybrid genetic background of C57BL/6 and C3H (B6C3F1) mice. Injected embryos were implanted into the oviducts of day-0.5 pseudo pregnant female Swiss Webster mice.

### Genotyping

Tails from the resulting pups were clipped 3 weeks after birth for genotype analysis. Genomic DNA was isolated by the DNeasy Tissue kit (QIAGEN, Valencia, CA). Presence of the transgene was confirmed by PCR amplification of a 486-bp fragment (Frag1) of the intron-myr-*PIK3CA* as well as a 324–bp fragment (Frag2) of the eGFP. Specific primers for eGFP were as follows: Frag1 forward primer: 5′-AGG CAC TGG GCA GGT AAG TAT, Frag1 reverse primer: 5′-CAT GTT TGA TGG TGA CGA GTG; Frag2 forward primer: 5′-CGA CAA CCA CTA CCT GAG CA, Frag2 reverse primer: 5′-TTA GGA AAG GAC AGT GGG AGT G. Genomic DNA (2 μl) was amplified in 25 μl of the PCR reaction containing 200 μmol/L each dNTP, 20 pmol of each primer, the standard buffer supplemented with 1.5 U *Taq* polymerase (Roche, Indianapolis, IN), and 1.5 mmol/L MgCl_2_. After initial denaturation at 94°C for 4 minutes, 30 cycles of PCR were performed with denaturation at 94°C for 15 seconds, annealing at 55°C for 20 seconds, and extension at 72°C for 45 seconds. The last extension was at 72°C for 7 minutes.

### RNA Isolation and Reverse Transcriptase-Polymerase Chain Reaction (RT-PCR)

Total RNA was isolated from 100 to 500 mg of fresh tissue or 1×10^6^ cultured cells with TRIzol reagent (Invitrogen). Total RNA was reverse-transcribed using the Superscript first-strand synthesis kit for RT-PCR (Invitrogen). Reverse-transcribed cDNA was amplified in 25 μl of the PCR reaction containing 200 μmol/L each dNTP, 20 pmol of each primer, the standard buffer supplemented with 1.5 U *Taq* polymerase (Roche), and 1.5 mmol/L MgCl_2_. After initial denaturation at 94°C for 4 minutes, 35 cycles of PCR were performed with denaturation at 94°C for 15 seconds, annealing at 55°C for 20 seconds, and extension at 72°C for 45 seconds. The last extension was at 72°C for 7 minutes. Specific primers for GFP were as follows: eGFP forward primer: 5′-AGC TGA CCC TGA AGT TCA TCT G, GFP reverse primer: 5′-GAT CTT GAA GTT CAC CTT GAT GC.

### Real-time RT-PCR

cDNA was quantified by real-time RT-PCR on the ABI Prism 7900 Sequence Detection System (Applied Biosystems, Foster City, CA). PCR was performed using Sybr Green PCR Core reagents (Applied Biosystems). PCR amplification of the housekeeping genes GAPDH was performed for each sample as a control for sample loading and to allow normalization among samples. A standard curve was constructed containing the *PIK3CA* cDNA and amplified by the real-time PCR. Each sample was run in duplicate and each PCR experiment included two non-template control wells.

### Laser capture microdissection (LCM)

LCM was performed as described by our previous study [48]. Briefly, cryosections (10 μm) from human or mouse ovaries were mounted on a polyethylene foil slide (SL Microtest, Jena, Germany). After rapid hematoxylin staining, sections were subjected to LCM utilizing the μCUT Laser-MicroBeam System (SL Microtest) with a fine ultraviolet laser, which enables the contact-free isolation of single cells or groups of cells. The microdissected cells were catapulted into the lid of a 0.5-ml reaction tube containing RNA isolation buffer. RNA was isolated by TRIzol reagent.

### Immunostaining

Immunohistochemical staining was performed using the avidin-biotin-peroxidase method [49]. Sections were pretreated with 0.03% H_2_O_2_ for 20 minutes to block endogenous peroxidase activity and incubated in matched normal sera (Vector Laboratories, Burlingame, CA). The following primary antibodies were used in this study: Cytokeratin (Hybridoma Bank, Iowa City, IA, 1∶200) rabbit anti-p110α (Cell Signaling, Danvers, MA 1∶200) and mouse anti-human p110a (Pharmigen, CA, 1∶200). Mouse on Mouse (M.O.M.) kit (Vector) was used for monoclonal antibody. The Vectastain ABC kit was applied as described by the manufacturer (Vector Laboratories). Sections were counterstained with Gill's hematoxylin (Vector Laboratories). Images were acquired through a Cool SNAP Pro color digital camera (Media Cybernetics).

### RNA interference/Transfection of Synthetic siRNA

Synthetic *SMART*pool siRNAs targeting mouse *PIK3CA* (Dharmacon, Chicago, IL) or appropriate si*CONTROL* non-targeting siRNAs (Dharmacon) were transfected into cultured cells. Transfection was performed using Lipofectamine^TM^2000 (Invitrogen) following the manufacturer's instructions. Forty-eight hrs post transfection, total RNA was extracted to examine the *PIK3CA* expression by real-time RT-PCR.

### *In vitro* Cell Transformation Assay

A soft agar assay was performed using Cell Transformation Detection Assay Kit (Chemicon, Temecula, CA) following the manufacturer's instructions [41].

### Statistics

Statistical analysis was performed using the SPSS statistics software package (SPSS, Chicago, IL). All results were expressed as mean±SD, and p<0.05 was used for significance.

## Supporting Information

Figure S1 (0.05 MB PDF)
