# Supplementary figures and images for: Expression of Activated PIK3CA in Ovarian Surface Epithelium Results in Hyperplasia but Not Tumor Formation
Source: PLoS One. 2009 Jan 27;4(1):e4295. doi: 10.1371/journal.pone.0004295 (PMC2629728; doi:10.1371/journal.pone.0004295)

Figure S1

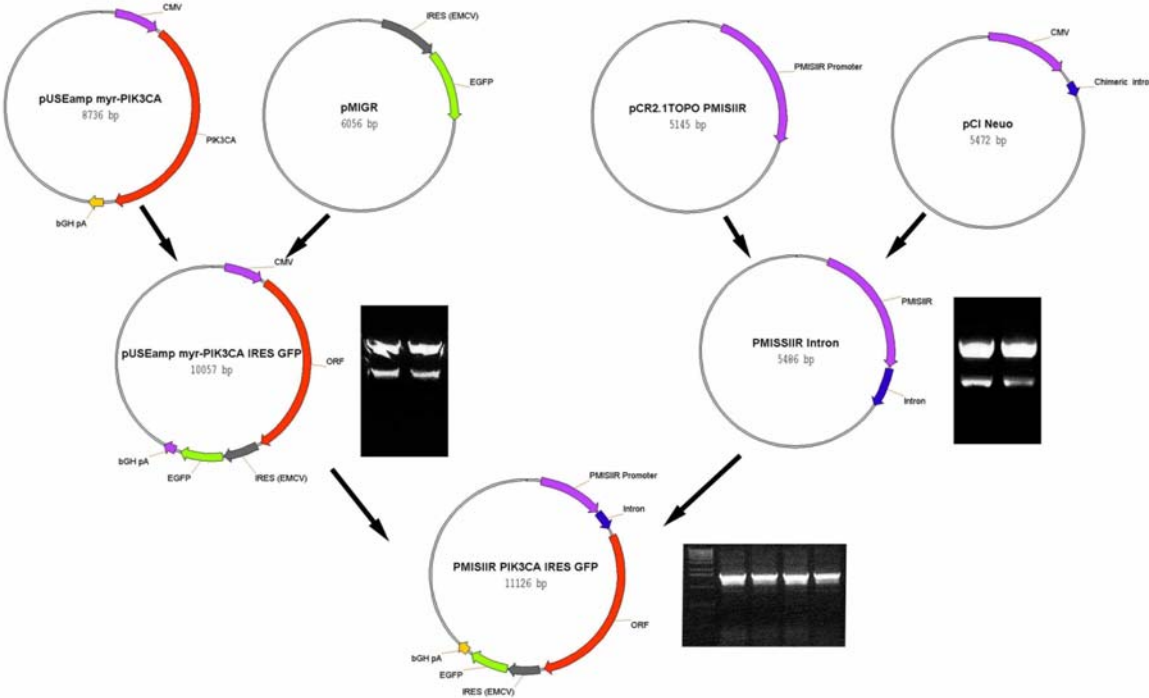

Supplement: Figure S1 — (0.05 MB PDF) [file pone.0004295.s001.pdf]
